# The Mount Vernon Hospital for Consumption

**Published:** 1903-10-31

**Authors:** 


### THE MOUNT VERNON HOSPITAL FOR CONSUMPTION.

The Marquis of Zetland, President of the Hospital, and a large concourse of Governors and subscribers attended the Mount Vernon Hospital on Tuesday, October 27th, on the occasion of the opening of the new East block.

After the singing of a hymn, and the offering of prayers by the chaplain, Mr. B. A. Lyon (the Chairman) introduced Lord Zetland, and remarked that during last year his lordship had consented to become President of the Hospital. The committee of management had asked him to do so, chiefly on account of the interest shown by Lord Zetland in the hospital for some time past by supporting a number of beds for the benefit of Yorkshiremen.

The Marquis of Zetland said that he felt it to be a high honour to be invited to take such a prominent part in so interesting a ceremony. Referring to the past history of the hospital, he remarked that it was founded in 1860, and began its work in Fitzroy Square, where there was simply accommodation for a few out-patients. The present building was commenced in 1880, when accommodation for 25 patients was provided. Nothing spoke more forcibly as to the efficient manner in which the work of the institution was carried out, and as to its popularity, than the fact that there were at the present time 270 patients, who had been medically examined and considered fit for admission into the hospital, waiting their turn for reception. Some of them were under treatment as out-patients at Fitzroy Square. The sum of £10,000 was still needed to defray the expenses connected with the extension, and he trusted that that sum would soon be forthcoming and that their friends and the public would come forward in such a way as to enable them shortly to make the announcement that they were free of debt. It was of the utmost importance that cases of consumption should be taken in hand promptly. He had been a subscriber for some years, for two reasons: first, because of the great admiration he felt for the institution, and also from his strong desire to confer what benefits he could on his fellow-men in Yorkshire. In illustration of the happy results that within his own knowledge had been obtained by patients at hospitals, he instanced two cases of Yorkshiremen who had both left the hospital three years ago, and neither of whom had had any return of the disease. These two men had, as far as possible, followed the advice given them as to open windows and diet. He felt great satisfaction in coming there and declaring the new block open, and trusted the hospital had a long life of usefulness in front of it, and he wished it success with all his heart.

Mr. B. A. Lyon, in proposing a vote of thanks to Lord Zetland, said that he himself felt the greatest satisfaction that he had lived to see what had been achieved. He had been for 20 years chairman of the hospital, through good times and bad times. The committee was an ideal one, and they never had a jarring note, but were all animated by one purpose, the carrying on of the hospital; supported by a generous public and by the able and devoted services of the medical staff they had made it second to none in London. All their success had been the work of the medical men, to whom they owed the highest tribute of praise. Not only was this hospital prepared for the reception of 145 patients, but they hoped that within six months from the present time the beautiful building at Northwood, providing accommodation for 100 more, would be ready. It was now rapidly nearing completion and would be the most beautiful building of its kind near London. They would then be able to provide for 245 patients altogether and it would be the second largest hospital of that kind in London.

The vote of thanks was seconded by Sir Hermann Weber, M.D., Consulting Physician to the hospital, who said that that hospital deserved the patronage of the public more than any other, because it was the first to adopt the open-air treatment of consumption. The committee of the hospital had always shown great spirit and courage; they had also shown their judgment in the selection of its position.

At the termination of the proceedings a gratifying incident occurred, when the clerk mentioned by Mr. Lyon rose in the body of the hall to offer his testimony to the benefits received by his wife from her treatment at that hospital, and said that four years ago he was told that it was only a question of time with her, but that she was then sitting beside him and was perfectly well and strong.

The present extension of the hospital cost £16,000. In addition to providing accommodation for 45 patients, it also contains service and staff rooms, providing on the top floor sleeping accommodation for five nurses and also a sitting-room and dining-room for the whole of the nursing staff. The south-west front has been constructed on the gallery principle, in order that patients may enjoy as much fresh air as possible. Care has been taken to provide arrangements whereby during severe weather adequate protection can be ensured.

The cost of the site and the whole of the buildings was £43,500. Of the 45 beds now provided the committee do not feel justified in opening more than 25, as they still need a sum of £10,000. They receive a grant of £1,000 per annum from the King's Hospital Fund, and are also well supported by the Hospital Sunday and Saturday Funds, but they need the continued help of their friends in order to liquidate the present deficiency and to ensure a steady annual income. The hospital has no invested property, and is dependent entirely upon annual subscriptions and donations.

A large out-patient department has been purchased in Fitzroy Square, and when this is completed, as it is hoped will be the case in about a month, the needs of the hospital in this particular will be fully supplied. The cost of this department is £6,000, part of which sum has been raised by a mortgage on the property.

